# Benefits of Stimulus Congruency for Multisensory Facilitation of Visual Learning

**DOI:** 10.1371/journal.pone.0001532

**Published:** 2008-01-30

**Authors:** Robyn S. Kim, Aaron R. Seitz, Ladan Shams

**Affiliations:** 1 Department of Psychology, University of California Los Angeles, Los Angeles, California, United States of America; 2 Department of Psychology, Boston University, Boston, Massachusetts, United States of America; Ecole Polytechnique Federale de Lausanne, Switzerland

## Abstract

**Background:**

Studies of perceptual learning have largely focused on unisensory stimuli. However, multisensory interactions are ubiquitous in perception, even at early processing stages, and thus can potentially play a role in learning. Here, we examine the effect of auditory-visual congruency on visual learning.

**Methodology/Principle Findings:**

Subjects were trained over five days on a visual motion coherence detection task with either congruent audiovisual, or incongruent audiovisual stimuli. Comparing performance on visual-only trials, we find that training with congruent audiovisual stimuli produces significantly better learning than training with incongruent audiovisual stimuli or with only visual stimuli.

**Conclusions/Significance:**

This advantage from stimulus congruency during training suggests that the benefits of multisensory training may result from audiovisual interactions at a perceptual rather than cognitive level.

## Introduction

Perceptual learning has been the subject of extensive study in recent years. This type of learning is particularly interesting because it seems to demonstrate a surprising degree of cortical plasticity even in adult primary visual [1]–[2], auditory [3] and somotosensory areas [4]. To date, most studies of perceptual learning have focused on the learning of sensory features with a single modality. However, crossmodal interactions are ubiquitous in human perception, and can occur even at early stages of processing in areas previously viewed as “sensory-specific” [5]–[11]. Furthermore, crossmodal interactions are known to play an important role in the development of perceptual systems [12]–[14]. Therefore, it is likely that crossmodal interactions can mediate perceptual learning in the mature brain as well.

To explore the possible benefit of multisensory interactions in adult perceptual learning, we recently compared multisensory with unisensory training [15] using a coherent motion detection and discrimination task. Compared to a visually trained (V) group, the audio-visually trained (AV) group showed faster learning on visual trials across the ten training sessions, suggesting that multisensory training promotes more effective encoding of information and/or better retention of learning than unisensory training. Additionally, the results of a direction test showed that performance was significantly greater for the trained than the untrained directions, indicating that the observed improvements reflected perceptual learning.

However, while this study demonstrated that sound could facilitate visual perceptual learning, it did not address the question of whether the directional congruency of the sound with the visual stimulus was a significant factor in the learning enhancement. Research by Seitz et al. [16] has shown that the presence of incongruent stimuli during training can interfere with learning. This raises the question of whether the presence of incongruent extramodal motion stimuli (for instance auditory motion in the opposite direction to visual motion) will facilitate or inhibit learning. In the current study, we compare the effect of congruent audiovisual training with that of incongruent audiovisual training. If the facilitatory effects are merely due to extra attention/arousal or information, then an incongruent motion stimulus should provide the same level of facilitation as do congruent motion stimuli, as it contains equal stimulus energy and provides the same task-information as the congruent sound. On the other hand, if the facilitation occurs at a perceptual level rather than a cognitive, decisional one, then one may expect inhibition between two motion signals of opposite directions.

In addition, this experiment rules out a few alternative explanations that the previous experiment did not address. Namely: A) The “visual-alone” trials that were used for comparing the performance of the AV group with the V group did contain sound (stationary noise), which could have caused enhanced attention for the AV group, and thus enabled superior performance. B) While the visual signal exposure was equal between the two groups, the AV group had additional “audio-alone” trials in their training. Longer training sessions and/or the interleaving of “auditory-alone” trials with “visual-alone” trials may have facilitated perceptual learning for the AV group. C) The task for the AV group was to detect and discriminate directional motion (regardless of modality), whereas the task for the V group was to detect and discriminate visual motion. It is possible that the multisensory task for the AV group was more demanding and engaged attention more strongly, and thus produced the improved learning. Crucially, the AV training sessions in the current experiment include true unimodal visual trials (without stationary sound) for comparison with V group performance; also, “audio-alone” trials have been eliminated, thus equating length of training sessions and spacing of unimodal trials for all groups. Finally, the addition of the incongruent AV group addresses the influence of task demands, as their task is identical to that of the congruent AV group. In summary, this study addresses the question of whether the facilitation of learning by sound is specific to situations in which the sound signal is congruent with visual signal.

## Methods

### Participants

Twenty-one paid subjects (aged 19–39) with normal or corrected-to-normal vision and normal hearing were recruited from the UCLA population and randomly assigned to congruent multisensory (n = 7), incongruent multisensory (n = 7), and unisensory (n = 7) groups. All participants gave informed consent for participation in the study, which was approved by the UCLA institutional review board.

### Task

**Figure 1:**
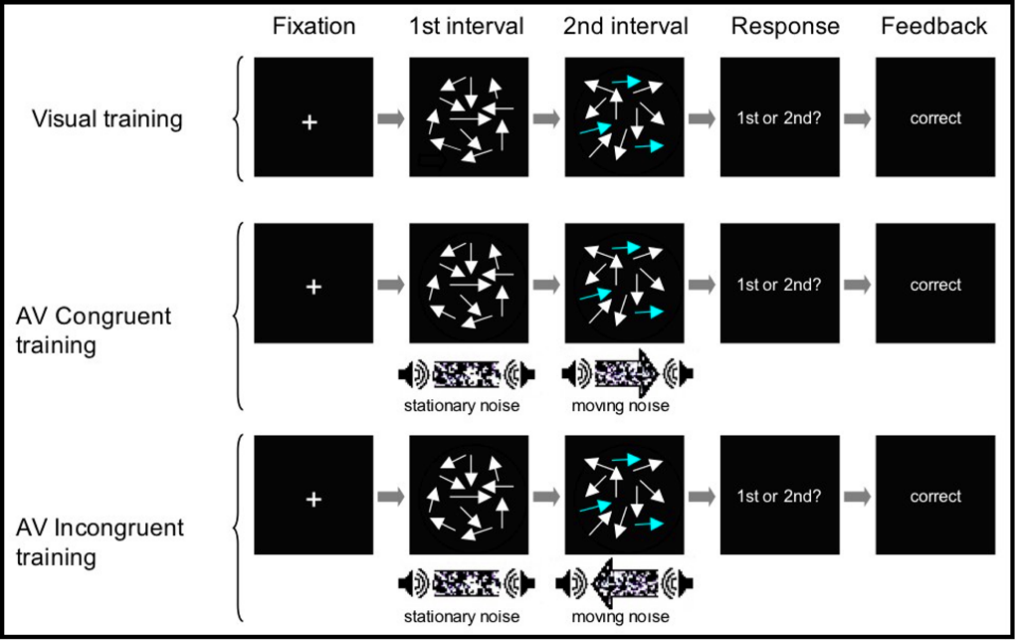
Task Schematic. Cartoon depiction of one trial for each training condition. Arrows indicate motion direction of dots, with coherently moving dots represented by blue colored arrows for illustration purposes (in the second interval for these examples). The top row shows one trial for the unisensory (Visual) group, the second row for the congruent multisensory group, and the third for the incongruent multisensory group.

The task consisted of a two-interval forced-choice task in which observers reported in which interval (first or second) they detected a directional stimulus by pressing keys on the keyboard (‘a’ for the first interval, ‘z’ for the second). As shown in Figure 1, each trial consisted of a sequence of two visual or audiovisual stimuli (described in more detail in Visual Stimuli and Auditory Stimuli below). In one interval, the stimulus contained directional motion, in the other it contained random motion. After presentation of the two intervals, participants were cued to respond whether they detected motion in the first or the second stimulus-interval. Participants were told they could answer based on either visual or auditory stimulus. Feedback was provided immediately after their response.

### Visual Stimuli

Visual stimuli were dynamic dot patterns of low motion coherence, presented at three levels of coherence tailored to each subject (see Stimulus Levels, below). A Movshon/Newsome-type motion algorithm [17] was employed with white dots (0.2 degree radius) in a 1°–10° annulus with a dot density of 16.7 dots per deg^2^/s and dot speed of 12 deg/s. In this motion algorithm, the subset of coherently moving dots is newly chosen in each frame, and the probability of a given dot lasting more than one frame is the same as the coherence level; positions of noncoherent dots are randomly generated for each frame. Because perception of cardinal directions may be robust to training [18], we chose to train 190° (instead of 180°) for leftward motion.

### Auditory Stimuli

We designed auditory motion stimuli to be analogous to the visual motion stimuli. Auditory motion was created by varying the amplitude of Gaussian white noise linearly (70–76 dB) between left and right speakers over 300 ms. This produced a percept of a stimulus moving left or right. These were presented at three signal-to-noise levels tailored to each subject (see Stimulus Levels, below) by masking the auditory signal with varying levels of white noise (bandwidth 2–10 KHz, butterworth filtered, ramped). Speakers were placed on the left and right side of the monitor with the midpoint between the speakers aligned with the fixation point. This produced the perception of sounds that were largely co-localized with the visual motion stimulus. Different coherence levels were created by varying the amplitude ratio between the auditory motion and the noise mask (i.e., computing a weighted average of auditory-motion signal and noise mask). Stimuli at all signal-to-noise levels were normalized so that each had the same root mean square and produced a reading of 76 dB on a sound-pressure meter.

### Stimulus Levels

Visual and auditory levels for each subject were determined through the examination of psychometric functions for each modality using data from the practice tests. For each subject, we chose levels that approximately corresponded to 55%–65%, 65%–75%, and 75%–85% correct detection. The mean coherence levels for the three groups did not significantly differ (congruent multisensory [low 4±.2, mid 8±.5, high 12±.8]; incongruent multisensory [low 4±.3, mid 7±.6, high 11±1]; unisensory [low 4±.3, mid 7±.8, high 11±1.1]).

### Procedure

For each participant, the experiment spanned eight days. All elements of the experiment were the same between groups except for training. The first day served primarily to acclimate the subjects to the task (and thus minimize task-learning effects during training) and to determine appropriate stimulus levels. This practice was composed of two tests consisting of unimodal stimuli (one audio and one visual) at high coherence (i.e. low difficulty) levels. The order of the two tests was counterbalanced across subjects. The second day consisted of a pre-test of four different visual motion directions (10°, 100°, 190°, 280°) without feedback. The last (eighth) day consisted of an identical post-test. Training sessions were conducted for 5 days (session numbers 3–7) and included feedback. The trained visual direction for all groups was leftward (190°). Training sessions for all groups lasted approximately an hour, and contained a total of 1200 trials: Visual training sessions consisted of three visual levels with 400 trials of each, and audiovisual training sessions consisted of 300 visual-only (silent) trials (three visual levels with 100 trials of each), and 900 audiovisual trials (three visual levels×three auditory levels with 100 trials of each). All trial types were pseudo-randomly interleaved. For the congruent audiovisual group, audiovisual trials always contained congruent directions across modalities (leftward auditory motion); for the incongruent multisensory group, audiovisual trials always contained opposing visual and auditory directions (rightward auditory motion). Within one audiovisual trial, the moving auditory stimulus was always in the same interval as the coherently moving visual stimulus. Coherence levels were not correlated between auditory and visual stimuli.

### Analysis

In all groups, the analysis is done on data obtained from trials consisting of visual stimuli only. In this way, performance is evaluated for identical trial types for each group. Also, for this study we are most concerned with long-lasting perceptual learning effects (i.e. performance improvements that are retained across days), as opposed to potential improvements within a session. Given this, we consider only the first third of trials in each session for data analysis (results are qualitatively similar for other early phases of the sessions and full sessions). In this way we are able to avoid potential contamination from effects of fast learning and deterioration [15].

## Results

**Figure 2:**
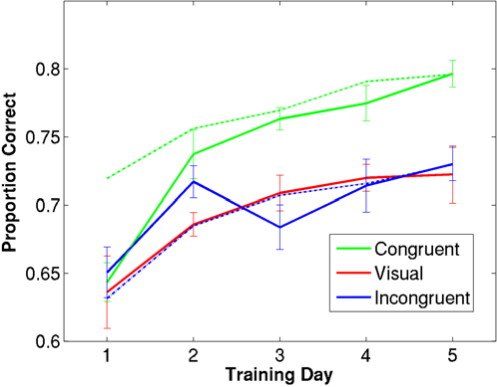
Data from each training session for congruent audiovisual group (green), unisensory visual group (red), and incongruent audiovisual group (blue). Ordinate is proportion correct averaged across three signal levels, abscissa reflects training session number. Solid lines reflect performance on visual-only trials over the first third of each session; dashed lines represent performance on audiovisual trials over the first third of each session. Error bars reflect within-group standard error [19].

In Figure 2, we show performance for the congruent audiovisual trained group (green), incongruent audiovisual trained group (blue), and the unisensory trained group (red), for visual-only trials (solid lines) and audiovisual trials (dashed lines) across the five days of training. While it is evident that there is a tendency for improvement in each group, improvement is clearly greatest for the congruent group. While the change in performance across the five days was highly significant for the congruent group (F(4,24) = 14.158, p<.0001, one-way repeated-measure ANOVA), the performance change was only moderately significant for the unisensory group (F(4,24) = 2.938, p = .04) and marginally significant for the incongruent group (F(4,24) = 2.937, p = .053). Furthermore a 3-way ANOVA (Training Day×Training Condition×Stimulus Level) shows a significant effect of training day (F(1,18) = 62.761, p<.01) and stimulus level (F(1,18) = 77.506, p<.01), and an interaction between training day and training condition between the first and last day of training (F(2,18) = 3.702, p<.05). However, we found no interaction between training day and stimulus level (F(2,26) = 1.144, p = .3299); therefore, we collapse data across stimulus levels.

While both congruent and vision groups improved significantly with training, the degree and rate of learning for the congruent audiovisual group surpassed that of the visual trained group; i.e., the congruent group followed a quadratic trajectory (polynomial regression analysis, F(1,32) = 8.67, p<.01) whereas the vision and incongruent groups followed a linear course (polynomial regression analysis, F(1,33) = 12.488, p<.001; F(1,33) = 7.5432, p = .01, respectively).

During training audiovisual trials were present, in addition to visual trials, in the congruent and incongruent groups (shown by dashed lines in Figure 2). For the congruent group, we found that performance on the audiovisual trials was significantly different than visual-only trials on the first day of training (paired t-test, p<.01), but this difference largely disappeared by the second day of training (paired t-test, p = .13). For the congruent group, we found that audiovisual performance never significantly differed from visual-only performance.

**Figure 3:**
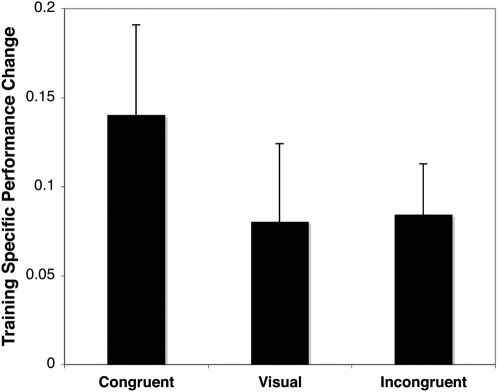
Difference in learning effect (i.e. increase in percent correct from pre- to post-test) between trained and untrained directions, for congruent, visual, and incongruent training groups. Error bars reflect standard error.

Tests of multiple motion directions, including the trained direction and three untrained directions, were conducted before and after training to examine the specificity of learning. We compared the change in performance from pre- to post-test for the trained direction with the average change for the three untrained directions. If indeed the learning reflects perceptual learning rather than (or in addition to) general task learning, the improvement should not be equal between trained and untrained directions. Figure 3 shows the difference in learning effect (i.e. increase in percent correct from pre- to post-test) between the trained and untrained directions for the audiovisual congruent, visual, and audiovisual incongruent trained groups, respectively. Similarly to performance during training, the congruent audiovisual trained group demonstrates a greater learning effect for the trained compared to untrained directions than the other two groups. The advantage for the trained direction after training is significant for the congruent group and the incongruent group (p<0.05, paired t-test), suggesting that the observed improvement in performance indeed reflects low-level perceptual learning. However, the unisensory group only showed a trend for greater improvement for the trained direction than untrained directions (p = 0.12).

## Discussion

Consistent with our previous study [15], we found superior learning in a visual task when subjects were trained with congruent audiovisual stimuli as compared to when subjects are trained with purely visual stimuli. This holds even when the length of the training sessions and the trial spacings are equated between groups, and when the visual-alone trials used for comparison between groups are physically identical (i.e., silent).

Furthermore, we found that training with an incongruent audiovisual stimulus failed to produce a similar facilitating effect during training. If the facilitation of learning was a result of enhanced general alerting caused by sound, then an enhancement of learning should have occurred for both the congruent and incongruent groups. The failure of the incongruent training paradigm to produce enhanced learning also argues against the explanation that the audiovisual condition produces better learning by being more demanding, and therefore more attentionally engaging than the visual alone condition, since both congruent and incongruent training contain audiovisual stimuli. If anything, incongruent training could be considered more difficult than congruent training, leading to the expectation that incongruent training would engage even more attention and produce better performance than congruent training. In actuality, since the task is one of motion detection and not of direction discrimination, the congruence of the audio and visual stimuli should not matter, as the subject need only detect any motion, regardless of direction, to choose the correct interval.

Another possibility is that the incongruent auditory stimuli did produce some level of alerting, but that these were countered by inhibitory interactions at the sensory level. For instance, inhibitory interactions at the sensory level are well established between opposite directions of visual motion [20]–[22]. Given that recent neuroimaging studies suggest brain areas specialized to process motion (such as MT) may process multisensory information [23], it seems likely that inhibitory interactions may also occur between crossmodal directional stimuli.

Performance on audiovisual trials differed between congruent and incongruent groups as well. As expected, congruent audiovisual trials yielded better detection than visual-only trials, but surprisingly, incongruent trials did not (see Figure 2), even though sound was equally informative for the task in both conditions. The fact that incongruent sound did not benefit performance on audiovisual trials, whereas congruent sound did, supports the conclusion that the auditory stimuli are not affecting performance at a decisional or cognitive level, but rather at a perceptual level. This differential performance on audiovisual trials may be responsible for the difference in visual learning between the congruent and incongruent trained groups. It is possible that the improved perception (as demonstrated by the enhanced performance) is the underlying factor for the benefit of multisensory training protocol. This would be consistent with the proposal that for learning to take place it requires that neuronal activity exceeds a threshold [24]. The multisensory stimulation causes such enhancement of neural activity (and hence the better performance) and thus facilitates learning when the sensory inputs are congruent, but not when they are incongruent.

Interestingly, while the congruent trained group initially performs worse on visual-only trials than audiovisual trials, this difference disappears with training and performance on the two trial-types becomes essentially equivalent. This convergence between visual-only and audiovisual performance suggests that crossmodal interactions are responsible for the facilitation of learning; i.e., that training with congruent audiovisual trials enhances visual processing such that the enhancements persist even when the auditory information is no longer present.

This study provides further evidence that multisensory training can affect visual learning at the perceptual level. While these results cannot speak to the exact mechanism by which multisensory training mediates visual learning, anatomical studies have found evidence for input to lower level visual areas both directly from primary and parabelt auditory cortex [25], [26], and also from the superior temporal polysensory area [25]. Thus, the congruent sound may facilitate visual learning through direct connections to visual areas, indirectly through multisensory areas, or through some combination of both. Much remains to be learned about the mechanisms of multisensory learning, but these results are a first step in parsing out how to capitalize on the dynamics of multisensory interactions to optimize learning paradigms.
